# Health risk assessment of heavy metals on PM_2.5_ in Tehran air, Iran

**DOI:** 10.1016/j.dib.2018.01.018

**Published:** 2018-01-31

**Authors:** Anoushiravan MohseniBandpi, Akbar Eslami, Mansour Ghaderpoori, Abbas Shahsavani, Ali Khani Jeihooni, Afshin Ghaderpoury, Abdolazim Alinejad

**Affiliations:** a Environmental and Occupational Hazards Control Research Center, Shahid Beheshti University of Medical Sciences, Tehran, Iran; b Nutritional Health Research Center, Lorestan University of Medical Sciences, Khorramabad, Iran; c Department of Environmental Health Engineering, School of Health and Nutrition, Lorestan University of Medical Sciences, Khorramabad, Iran; d Department of Environmental Health Engineering, School of Public Health, Shahid Beheshti University of Medical Sciences, Tehran, Iran; e Department of Public Health, Fasa University of Medical Sciences, Fasa, Iran; f Students Research Committee, Shahid Beheshti University of Medical Sciences, Tehran, Iran

**Keywords:** Air pollution, Health risk assessment, Heavy metals, Tehran

## Abstract

The general goal of this study was to measure the concentration of heavy metals on suspended particles and evaluate the health-risk assessment of these metals on human health. In this study, the concentration of heavy metals adsorbed on suspended particles less than 2.5 μm was determined. For determining of health-risk assessment, the developed method of USEPA was used. The health-risk assessment of carcinogen and non- carcinogen of suspended particles were evaluated in three main paths include oral ingestion, inhalation, and dermal contact. The average annual concentrations of Al, Fe, As, Cd, Cr, Cu, Mn, Ni, Pb, V, and Zn were 1.77, 1.14, 0.03, 0.02, 0.07, 0.15, 0.06, 0.03, 0.1, 0.04, and 0.14 μg/m^3^, respectively. Between different stations, the order for the heavy metals was followed as urban>traffic>suburban. The average risk of carcinogenic at urban stations in the spring for As, Cd, and Cr was 2.25*10^−9^, 2.09*10^–12^, and 2.05*10^–11^, respectively.

**Specifications Table**

**Table:** 

Subject area	*Chemistry*
More specific subject area	*Air monitoring and quality, health-risk assessment*
Type of data	*Table, figure*
How data was acquired	*Inductively coupled plasma atomic emission spectroscopy ICP-AES*
Data format	*Raw, analyzed,*
Experimental factors	*Measuring the heavy metals concentration (Al, Fe, As, Cd, Cr, Cu, Mn, Ni, Pb, V, and Zn) in PM*_*2.5*_*of air pollution of Tehran city. After determining the concentration, the health-risk assessment was calculated.*
Experimental features	*At present, Tehran city has 21 active stations for measuring and monitoring air pollutants. There are three types of stations:traffic (3 stations), urban (16 stations), and suburban (2 stations).*
Data source location	*Tehran city, Iran (35°34*′*-35°50*′*N and 51°08*′*-51°37*′*E)*
Data accessibility	*Data are included in this research and supplemented excel file*

**Value of the Data**

• Tehran is one of the most polluted cities in the world in terms of air pollution. The inhabitants of this city are always exposed to various air pollutants.
• According to studies, the most important source of air pollution in the city are suspended particles (especially PM_2.5_).
• Various compounds, such as heavy metals, can be absorbed on the particles so the assessment of health-risk assessment of pollutants is very important.
• The data of this study can be used for by to improve air qualityurban authorities

## 1 Data

Tehran is capital of Iran and according to the latest population census in Iran, in 2016, the population is over 11million people. The surface area of Tehran, 35°34′–35°50′N and 51°08′–51°37′E, is about 730 km^2^.

## 2 Experimental design, materials, and methods

**Fig. 1:**
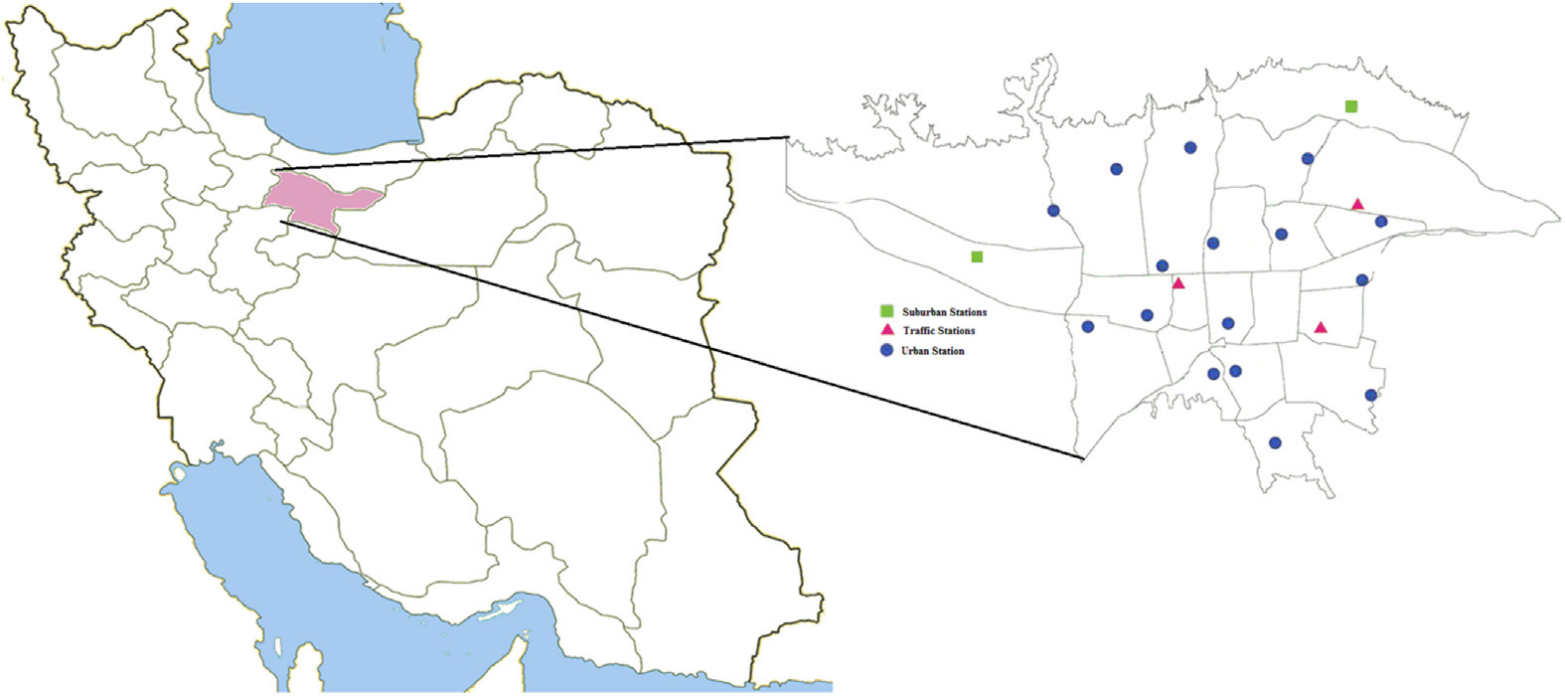
The location of monitoring stations of air pollution in Tehran city.

The general goal of this study was to measure the concentration of heavy metals on suspended particles and evaluate the health-risk assessment of these metals on human health. In this study, the concentration of heavy metals adsorbed on suspended particles less than 2.5 μm, PM_2.5_, was determined. The fiberglass filters (ID 460130, 30^mm^×31^mm^, Met One Instruments, USA) of Tehran Air Quality Control Company (TAQCC) were used to extract heavy metals from PM_2.5_. Extracted heavy metals were Al, Fe, As, Cd, Cr, Cu, Mn, Ni, V, and Zn. At present, Tehran has 21 active stations for measuring and monitoring air pollutants. In Tehran, there are three types of stations: traffic (3 stations), urban (16 stations), and suburban (2 stations). The location of the measurement and monitoring stations is shown in Fig. 1. The beta-attenuation monitor was used to measure PM_2.5_ particles. The air flow to the beta-attenuation monitor was 16.1 L/min. For the extraction of heavy metals from PM_2.5_ particles, at first, the fiberglass filters (after initial washing with distilled water, HCl, and HNO_3_ to remove impurities) were placed at 105 °C for 2 hours in the oven. Then, a mixture of HNO_3_, HClO_4_, and HCl was added into a Teflon container to digest the filter at 170 °C for 4 hours. In order to remove the residual acids, the Teflon container was placed on a heater at 95 to 100 °C. Finally, inductively coupled plasma atomic emission spectroscopy ICP-AES was used to measure the heavy metals concentration [1], [2]. For determining of health-risk assessment, the developed method of USEPA was used. Based on USEPA method, there are three main ways to intake dose include oral ingestion, inhalation, and dermal contact:

(1) $$ADD_{ing}=\frac{C*IRing*F*EF*ED*CF}{BW*AT}$$

(2) $$ADD_{inh}=\frac{C*IRing*F*EF*ED}{PEF*BW*AT}$$

(3) $$ADD_{der}=\frac{C*CF*SA*AF*ABS*F*EF*ED}{BW*AT}$$

Where, ADD_ing_, ADD_inh_, and ADD_der_ are the adsorbed dose of exposure to heavy metals via oral ingestion, inhalation, and dermal contact, respectively. C is heavy metals concentration on adsorbed PM_2.5_ (in term of mg/kg). ABS is absorption factor and value of carcinogenic and non-carcinogenic effects is 0.01 (without unit). AF is Adherence Factor (mg. cm^2^) and value of carcinogenic and non-carcinogenic effects is 0.07. AT is averaging time (in term of days) and value of carcinogenic and non-carcinogenic effects is 70*365 and ED*365, respectively. ED is exposure duration and value of carcinogenic and non-carcinogenic effects is 50 and 40 years, respectively. BW is body weight and is equal to 70 kg. CF is conversion factor and value of carcinogenic and non-carcinogenic effects is 0.000001 kg/mg. EF is exposure frequency and value of carcinogenic and non-carcinogenic effects is 250 day/years. F is a fraction of time spent at station in a day and value of carcinogenic and non-carcinogenic effects is 0.0694. PEF is particle emission factor and value of carcinogenic and non-carcinogenic effects is 1,360,000,000 m^3^/kg. SA is exposed skin surface area and value of carcinogenic and non-carcinogenic effects is 4350 cm^2^/day. IR_ing_ and IR_inh_ are ingestion and inhalation rate, respectively. IR_ing_ and IR_inh_ are 100 and 20, respectively [3], [4], [5]. After calculating of ADD, Hazard quotients, HQ, was determined. To estimate non-carcinogenic risk, HQ is determined according to Eq. (4):

(4) $$HQ=\frac{ADD_{ing,\;inh,\;or\;der}}{RfD_{ing,\;inh,\;or\;der}}$$

Where, HQ is hazard quotient. ADD is the exposure dose determined by Eqs. (1), (2), (3). The RfD is reference doses of each heavy metal. If HQ is less than one, the conditions are safe. After that, the hazard index, HI, was calculated. HI is used to assess the final non-carcinogenic risk posed by more than one heavy metal according to Eq. (5):

(5) $$HI=\sum_{i=1}^{n}HQ_{i}$$

Total Hazard Index refers to the sum of more than one HI for multiple pathways, oral ingestion, inhalation, and dermal contact, which is calculated according to Eq. (6):

(6) $$HI_{total}=HI_{ing}+HI_{inh}+HI_{der}$$

IF Hazard Index is less than one, there is no significant risk of the non-cancer effect. IF Hazard Index is more than one, there is a chance which non-cancer effects may occur [5], [6], [7], [8]. Table 1 shows the average concentration of heavy metals measured in urban, traffic, and suburban stations. The average-annual concentrations of aluminum, iron, arsenic, cadmium, chromium, copper, manganese, nickel, lead, vanadium and zinc were 1.77, 1.14, 0.03, 0.02, 0.07, 0.15, 0.06, 0.03, 0.1, 0.04, and 0.14 μg/m^3^, respectively. Among the different seasons, the highest and lowest heavy metals concentrations were related to aluminium and cadmium, respectively. Also, between different stations, the order for the heavy metals was followed as urban>traffic>suburban. The results of health-risk assessment include the adsorbed dose of exposure, hazard quotient, and a hazard index of heavy metals via three main pathways shown in Table 2, Table 3, Table 4, Table 5. The order of daily-exposure dose of metals at the stations was urban>traffic>suburban. The highest daily-exposure dose at urban, traffic, and suburban stations were related to Pb, Mn, and Ni, respectively. The highest HQ_total_ at urban, traffic, and suburban stations were related to As, Cd, and Pb, respectively. The maximum HI value, in spring, of at urban, traffic, and suburban stations were related to 1.35*10^−5^, 1.31*10^−5^, and 4.95*10^−6^, respectively. According to measured heavy metals, the carcinogenic risk for As, Cd, and Cr was calculated. The average carcinogenic risk at urban stations in the spring for As, Cd, and Cr was 2.25*10^−9^, 2.09*10^–12^, and 2.05*10^–11^, respectively.

**Table 1:** The concentrations of heavy metals (μg/m^3^) in the study area.

	**Spring**			**Summer**			**Autumn**			**Winter**		
	**Urban**	**Traffic**	**Suburban**	**Urban**	**Traffic**	**Suburban**	**Urban**	**Traffic**	**Suburban**	**Urban**	**Traffic**	**Suburban**
**Al**	2.020	1.620	1.480	2.890	2.280	2.160	1.610	1.840	1.360	1.860	1.290	0.880
**Fe**	1.790	1.440	1.060	1.840	1.270	0.620	0.950	0.940	0.640	1.310	1.120	0.710
**As**	0.030	0.020	0.007	0.030	0.023	0.005	0.031	0.036	0.007	0.060	0.030	0.031
**Cd**	0.048	0.050	0.002	0.015	0.028	0.002	0.021	0.032	0.002	0.034	0.031	0.020
**Cr**	0.070	0.080	0.020	0.080	0.080	0.025	0.140	0.110	0.072	0.070	0.070	0.081
**Cu**	0.280	0.150	0.039	0.230	0.130	0.040	0.180	0.220	0.037	0.340	0.110	0.040
**Mn**	0.090	0.110	0.120	0.070	0.060	0.050	0.050	0.040	0.043	0.070	0.030	0.032
**Ni**	0.030	0.040	0.014	0.050	0.020	0.023	0.037	0.040	0.020	0.032	0.057	0.028
**Pb**	0.120	0.160	0.130	0.090	0.160	0.040	0.150	0.013	0.130	0.190	0.020	0.050
**V**	0.030	0.050	0.019	0.041	0.050	0.013	0.050	0.050	0.030	0.040	0.050	0.020
**Zn**	0.09	0.17	0.018	0.15	0.22	0.021	0.16	0.19	0.32	0.19	0.12	0.08

**Table 2:** The adsorbed dose of exposure, hazard quotient, and hazard index of heavy metals via three main pathways in spring.

**Elements**	**Spring**			**AD**_**ing**_			**AD**_**inh**_		
	**urban**	**traffic**	**suburban**	**urban**	**traffic**	**suburban**	**urban**	**traffic**	**suburban**
**Al**	2.02E+00	1.62E+00	1.48E+00	1.37E-07	1.10E-07	1.01E-07	2.02E-11	1.62E-11	1.48E-11
**Fe**	1.79E+00	1.44E+00	1.06E+00	1.22E-07	9.78E-08	7.20E-08	1.79E-11	1.44E-11	1.06E-11
**As**	3.00E-02	2.00E-02	7.00E-03	2.04E-09	1.36E-09	4.75E-10	3.00E-13	2.00E-13	6.99E-14
**Cd**	4.80E-02	5.00E-02	2.00E-03	3.26E-09	3.40E-09	1.36E-10	4.79E-13	4.99E-13	2.00E-14
**Cr**	7.00E-02	8.00E-02	2.00E-02	4.75E-09	5.43E-09	1.36E-09	6.99E-13	7.99E-13	2.00E-13
**Cu**	2.80E-01	1.50E-01	3.90E-02	1.90E-08	1.02E-08	2.65E-09	2.80E-12	1.50E-12	3.89E-13
**Mn**	9.00E-02	1.10E-01	1.20E-01	6.11E-09	7.47E-09	8.15E-09	8.99E-13	1.10E-12	1.20E-12
**Ni**	3.00E-02	4.00E-02	1.40E-02	2.04E-09	2.72E-09	9.51E-10	3.00E-13	3.99E-13	1.40E-13
**Pb**	1.20E-01	1.60E-01	1.30E-01	8.15E-09	1.09E-08	8.83E-09	1.20E-12	1.60E-12	1.30E-12
**V**	3.00E-02	5.00E-02	1.90E-02	2.04E-09	3.40E-09	1.29E-09	3.00E-13	4.99E-13	1.90E-13
**Zn**	9.00E-02	1.70E-01	1.80E-02	6.11E-09	1.15E-08	1.22E-09	8.99E-13	1.70E-12	1.80E-13
**Elements**				**HQ ing**			**HQ inh**		
	**urban**	**traffic**	**suburban**	**urban**	**traffic**	**suburban**	**urban**	**traffic**	**suburban**
**Al**	2.02E+00	1.62E+00	1.48E+00	–	–	–	–	–	–
**Fe**	1.79E+00	1.44E+00	1.06E+00	–	–	–	–	–	–
**As**	3.00E-02	2.00E-02	7.00E-03	5.09E-06	3.40E-06	1.19E-06	7.49E-10	4.99E-10	1.75E-10
**Cd**	4.80E-02	5.00E-02	2.00E-03	3.26E-06	3.40E-06	1.36E-07	4.79E-10	4.99E-10	2.00E-11
**Cr**	7.00E-02	8.00E-02	2.00E-02	1.58E-06	1.81E-06	4.53E-07	2.44E-08	2.79E-08	6.98E-09
**Cu**	2.80E-01	1.50E-01	3.90E-02	4.75E-07	2.55E-07	6.62E-08	6.96E-11	3.73E-11	9.69E-12
**Mn**	9.00E-02	1.10E-01	1.20E-01	1.30E-07	1.59E-07	1.73E-07	6.42E-08	7.85E-08	8.56E-08
**Ni**	3.00E-02	4.00E-02	1.40E-02	1.02E-07	1.36E-07	4.75E-08	1.45E-11	1.94E-11	6.79E-12
**Pb**	1.20E-01	1.60E-01	1.30E-01	2.33E-06	3.10E-06	2.52E-06	3.42E-10	4.57E-10	3.71E-10
**V**	3.00E-02	5.00E-02	1.90E-02	4.04E-07	6.74E-07	2.56E-07	4.28E-11	7.13E-11	2.71E-11
**Zn**	9.00E-02	1.70E-01	1.80E-02	2.04E-08	3.85E-08	4.07E-09	3.00E-12	5.66E-12	5.99E-13
HI	-	-	-	1.34E-05	1.30E-05	4.85E-06	9.03E-08	1.08E-07	9.32E-08

**Elements**	**AD**_**inh**_			**AD**_**der**_			**AD**_**total**_		
	**urban**	**traffic**	**suburban**	**urban**	**traffic**	**suburban**	**urban**	**traffic**	**suburban**
**Al**	1.44E-11	1.16E-11	1.06E-11	4.18E-09	3.35E-09	3.06E-09	1.41E-07	1.13E-07	1.04E-07
**Fe**	1.28E-11	1.03E-11	7.56E-12	3.70E-09	2.98E-09	2.19E-09	1.25E-07	1.01E-07	7.42E-08
**As**	2.14E-13	1.43E-13	4.99E-14	6.20E-11	4.14E-11	1.45E-11	2.10E-09	1.40E-09	4.90E-10
**Cd**	3.42E-13	3.57E-13	1.43E-14	9.93E-11	1.03E-10	4.14E-12	3.36E-09	3.50E-09	1.40E-10
**Cr**	4.99E-13	5.71E-13	1.43E-13	1.45E-10	1.65E-10	4.14E-11	4.90E-09	5.60E-09	1.40E-09
**Cu**	2.00E-12	1.07E-12	2.78E-13	5.79E-10	3.10E-10	8.06E-11	1.96E-08	1.05E-08	2.73E-09
**Mn**	6.42E-13	7.85E-13	8.56E-13	1.86E-10	2.27E-10	2.48E-10	6.30E-09	7.70E-09	8.40E-09
**Ni**	2.14E-13	2.85E-13	9.99E-14	6.20E-11	8.27E-11	2.89E-11	2.10E-09	2.80E-09	9.80E-10
**Pb**	8.56E-13	1.14E-12	9.27E-13	2.48E-10	3.31E-10	2.69E-10	8.40E-09	1.12E-08	9.10E-09
**V**	2.14E-13	3.57E-13	1.36E-13	6.20E-11	1.03E-10	3.93E-11	2.10E-09	3.50E-09	1.33E-09
**Zn**	6.42E-13	1.21E-12	1.28E-13	1.86E-10	3.52E-10	3.72E-11	6.30E-09	1.19E-08	1.26E-09
**Elements**	**HQ der**			**HQ total**			**RI**		
	**urban**	**traffic**	**suburban**	**urban**	**traffic**	**suburban**	**urban**	**traffic**	**suburban**
**Al**	–	–	–	–	–	–	–	–	–
**Fe**	–	–	–	–	–	–	–	–	–
**As**	1.74E-09	1.16E-09	4.06E-10	5.10E-06	3.40E-06	1.19E-06	2.25E-12	1.50E-12	5.25E-13
**Cd**	3.42E-08	3.57E-08	1.43E-09	3.29E-06	3.43E-06	1.37E-07	2.09E-12	2.18E-12	8.70E-14
**Cr**	8.32E-09	9.51E-09	2.38E-09	1.62E-06	1.85E-06	4.62E-07	2.05E-11	2.34E-11	5.85E-12
**Cu**	1.66E-10	8.92E-11	2.32E-11	4.76E-07	2.55E-07	6.62E-08	–	–	–
**Mn**	2.79E-10	3.41E-10	3.72E-10	1.95E-07	2.38E-07	2.59E-07	–	–	–
**Ni**	3.96E-11	5.28E-11	1.85E-11	1.02E-07	1.36E-07	4.76E-08	–	–	–
**Pb**	1.63E-09	2.17E-09	1.77E-09	2.33E-06	3.11E-06	2.52E-06	–	–	–
**V**	3.06E-09	5.09E-09	1.94E-09	4.07E-07	6.79E-07	2.58E-07	–	–	–
**Zn**	1.07E-11	2.02E-11	2.14E-12	2.04E-08	3.85E-08	4.08E-09	–	–	–
HI	4.95E-08	5.41E-08	8.33E-09	1.35E-05	1.31E-05	4.95E-06	–	–	–

**Table 3:** The adsorbed dose of exposure, hazard quotient, and Hazard Index of heavy metals via three main pathways in summer.

**Elements**	**Summer**			**AD**_**ing**_			**AD**_**inh**_		
	**urban**	**traffic**	**suburban**	**urban**	**traffic**	**suburban**	**urban**	**traffic**	**suburban**
**Al**	2.89E+00	2.28E+00	2.16E+00	1.96E-07	1.55E-07	1.47E-07	2.89E-11	2.28E-11	2.16E-11
**Fe**	1.84E+00	1.27E+00	6.20E-01	1.25E-07	8.62E-08	4.21E-08	1.84E-11	1.27E-11	6.19E-12
**As**	3.00E-02	2.30E-02	5.00E-03	2.04E-09	1.56E-09	3.40E-10	3.00E-13	2.30E-13	4.99E-14
**Cd**	1.50E-02	2.80E-02	2.00E-03	1.02E-09	1.90E-09	1.36E-10	1.50E-13	2.80E-13	2.00E-14
**Cr**	8.00E-02	8.00E-02	2.50E-02	5.43E-09	5.43E-09	1.70E-09	7.99E-13	7.99E-13	2.50E-13
**Cu**	2.30E-01	1.30E-01	4.00E-02	1.56E-08	8.83E-09	2.72E-09	2.30E-12	1.30E-12	3.99E-13
**Mn**	7.00E-02	6.00E-02	5.00E-02	4.75E-09	4.07E-09	3.40E-09	6.99E-13	5.99E-13	4.99E-13
**Ni**	5.00E-02	2.00E-02	2.30E-02	3.40E-09	1.36E-09	1.56E-09	4.99E-13	2.00E-13	2.30E-13
**Pb**	9.00E-02	1.60E-01	4.00E-02	6.11E-09	1.09E-08	2.72E-09	8.99E-13	1.60E-12	3.99E-13
**V**	4.10E-02	5.00E-02	1.30E-02	2.78E-09	3.40E-09	8.83E-10	4.09E-13	4.99E-13	1.30E-13
**Zn**	1.50E-01	2.20E-01	2.10E-02	1.02E-08	1.49E-08	1.43E-09	1.50E-12	2.20E-12	2.10E-13
**Elements**				**HQ**_**ing**_			**HQ**_**inh**_		
	**urban**	**traffic**	**suburban**	**urban**	**traffic**	**suburban**	**urban**	**traffic**	**suburban**
**Al**	2.89E+00	2.28E+00	2.16E+00	–	–	–	–	–	–
**Fe**	1.84E+00	1.27E+00	6.20E-01	–	–	–	–	–	–
**As**	3.00E-02	2.30E-02	5.00E-03	5.09E-06	3.90E-06	8.49E-07	7.49E-10	5.74E-10	1.25E-10
**Cd**	1.50E-02	2.80E-02	2.00E-03	1.02E-06	1.90E-06	1.36E-07	1.50E-10	2.80E-10	2.00E-11
**Cr**	8.00E-02	8.00E-02	2.50E-02	1.81E-06	1.81E-06	5.66E-07	2.79E-08	2.79E-08	8.73E-09
**Cu**	2.30E-01	1.30E-01	4.00E-02	3.90E-07	2.21E-07	6.79E-08	5.71E-11	3.23E-11	9.94E-12
**Mn**	7.00E-02	6.00E-02	5.00E-02	1.01E-07	8.67E-08	7.22E-08	4.99E-08	4.28E-08	3.57E-08
**Ni**	5.00E-02	2.00E-02	2.30E-02	1.70E-07	6.79E-08	7.81E-08	2.42E-11	9.70E-12	1.11E-11
**Pb**	9.00E-02	1.60E-01	4.00E-02	1.75E-06	3.10E-06	7.76E-07	2.57E-10	4.57E-10	1.14E-10
**V**	4.10E-02	5.00E-02	1.30E-02	5.52E-07	6.74E-07	1.75E-07	5.85E-11	7.13E-11	1.85E-11
**Zn**	1.50E-01	2.20E-01	2.10E-02	3.40E-08	4.98E-08	4.75E-09	4.99E-12	7.32E-12	6.99E-13
**HI**	-	-	-	1.09E-05	1.18E-05	2.72E-06	7.92E-08	7.22E-08	4.47E-08

**Elements**	**AD**_**inh**_			**AD**_**der**_			**AD**_**total**_		
	**urban**	**traffic**	**suburban**	**urban**	**traffic**	**suburban**	**urban**	**traffic**	**suburban**
**Al**	2.06E-11	1.63E-11	1.54E-11	5.98E-09	4.71E-09	4.47E-09	2.02E-07	1.60E-07	1.51E-07
**Fe**	1.31E-11	9.06E-12	4.42E-12	3.80E-09	2.63E-09	1.28E-09	1.29E-07	8.89E-08	4.34E-08
**As**	2.14E-13	1.64E-13	3.57E-14	6.20E-11	4.76E-11	1.03E-11	2.10E-09	1.61E-09	3.50E-10
**Cd**	1.07E-13	2.00E-13	1.43E-14	3.10E-11	5.79E-11	4.14E-12	1.05E-09	1.96E-09	1.40E-10
**Cr**	5.71E-13	5.71E-13	1.78E-13	1.65E-10	1.65E-10	5.17E-11	5.60E-09	5.60E-09	1.75E-09
**Cu**	1.64E-12	9.27E-13	2.85E-13	4.76E-10	2.69E-10	8.27E-11	1.61E-08	9.10E-09	2.80E-09
**Mn**	4.99E-13	4.28E-13	3.57E-13	1.45E-10	1.24E-10	1.03E-10	4.90E-09	4.20E-09	3.50E-09
**Ni**	3.57E-13	1.43E-13	1.64E-13	1.03E-10	4.14E-11	4.76E-11	3.50E-09	1.40E-09	1.61E-09
**Pb**	6.42E-13	1.14E-12	2.85E-13	1.86E-10	3.31E-10	8.27E-11	6.30E-09	1.12E-08	2.80E-09
**V**	2.92E-13	3.57E-13	9.27E-14	8.48E-11	1.03E-10	2.69E-11	2.87E-09	3.50E-09	9.10E-10
**Zn**	1.07E-12	1.57E-12	1.50E-13	3.10E-10	4.55E-10	4.34E-11	1.05E-08	1.54E-08	1.47E-09
**Elements**	**HQ**_**der**_			**HQ**_**total**_			**RI**		
	**urban**	**traffic**	**suburban**	**urban**	**traffic**	**suburban**	**urban**	**traffic**	**suburban**
**Al**	–	–	–	–	–	–	–	–	–
**Fe**	–	–	–	–	–	–	–	–	–
**As**	1.74E-09	1.33E-09	2.90E-10	5.10E-06	3.91E-06	8.49E-07	2.25E-12	1.72E-12	3.75E-13
**Cd**	1.07E-08	2.00E-08	1.43E-09	1.03E-06	1.92E-06	1.37E-07	6.53E-13	1.22E-12	8.70E-14
**Cr**	9.51E-09	9.51E-09	2.97E-09	1.85E-06	1.85E-06	5.78E-07	2.34E-11	2.34E-11	7.31E-12
**Cu**	1.37E-10	7.73E-11	2.38E-11	3.91E-07	2.21E-07	6.79E-08	–	–	–
**Mn**	2.17E-10	1.86E-10	1.55E-10	1.51E-07	1.30E-07	1.08E-07	–	–	–
**Ni**	6.60E-11	2.64E-11	3.04E-11	1.70E-07	6.79E-08	7.81E-08	–	–	–
**Pb**	1.22E-09	2.17E-09	5.43E-10	1.75E-06	3.11E-06	7.77E-07	–	–	–
**V**	4.18E-09	5.09E-09	1.32E-09	5.57E-07	6.79E-07	1.76E-07	–	–	–
**Zn**	1.78E-11	2.62E-11	2.50E-12	3.40E-08	4.98E-08	4.76E-09	–	–	–
**HI**	2.78E-08	3.84E-08	6.77E-09	1.10E-05	1.19E-05	2.78E-06	–	–	–

**Table 4:** The adsorbed dose of exposure, hazard quotient, and Hazard Index of heavy metals via three main pathways in autumn.

	**Autumn**			**ADi**_**ng**_			**AD**_**inh**_		
	**urban**	**traffic**	**suburban**	**urban**	**traffic**	**suburban**	**urban**	**traffic**	**suburban**
**Al**	1.61E+00	1.84E+00	1.36E+00	1.09E-07	1.25E-07	9.24E-08	1.61E-11	1.84E-11	1.36E-11
**Fe**	9.50E-01	9.40E-01	6.40E-01	6.45E-08	6.38E-08	4.35E-08	9.49E-12	9.39E-12	6.39E-12
**As**	3.10E-02	3.60E-02	7.00E-03	2.11E-09	2.44E-09	4.75E-10	3.10E-13	3.60E-13	6.99E-14
**Cd**	2.10E-02	3.20E-02	2.00E-03	1.43E-09	2.17E-09	1.36E-10	2.10E-13	3.20E-13	2.00E-14
**Cr**	1.40E-01	1.10E-01	7.20E-02	9.51E-09	7.47E-09	4.89E-09	1.40E-12	1.10E-12	7.19E-13
**Cu**	1.80E-01	2.20E-01	3.70E-02	1.22E-08	1.49E-08	2.51E-09	1.80E-12	2.20E-12	3.69E-13
**Mn**	5.00E-02	4.00E-02	4.30E-02	3.40E-09	2.72E-09	2.92E-09	4.99E-13	3.99E-13	4.29E-13
**Ni**	3.70E-02	4.00E-02	2.00E-02	2.51E-09	2.72E-09	1.36E-09	3.69E-13	3.99E-13	2.00E-13
**Pb**	1.50E-01	1.30E-02	1.30E-01	1.02E-08	8.83E-10	8.83E-09	1.50E-12	1.30E-13	1.30E-12
**V**	5.00E-02	5.00E-02	3.00E-02	3.40E-09	3.40E-09	2.04E-09	4.99E-13	4.99E-13	3.00E-13
**Zn**	1.60E-01	1.90E-01	3.20E-01	1.09E-08	1.29E-08	2.17E-08	1.60E-12	1.90E-12	3.20E-12
				**HQ**_**ing**_			**HQ**_**inh**_		
	**urban**	**traffic**	**suburban**	**urban**	**traffic**	**suburban**	**urban**	**traffic**	**suburban**
**Al**	1.61E+00	1.84E+00	1.36E+00	–	–	–	–	–	–
**Fe**	9.50E-01	9.40E-01	6.40E-01	–	–	–	–	–	–
**As**	3.10E-02	3.60E-02	7.00E-03	5.26E-06	6.11E-06	1.19E-06	7.74E-10	8.99E-10	1.75E-10
**Cd**	2.10E-02	3.20E-02	2.00E-03	1.43E-06	2.17E-06	1.36E-07	2.10E-10	3.20E-10	2.00E-11
**Cr**	1.40E-01	1.10E-01	7.20E-02	3.17E-06	2.49E-06	1.63E-06	4.89E-08	3.84E-08	2.51E-08
**Cu**	1.80E-01	2.20E-01	3.70E-02	3.06E-07	3.73E-07	6.28E-08	4.47E-11	5.47E-11	9.19E-12
**Mn**	5.00E-02	4.00E-02	4.30E-02	7.22E-08	5.78E-08	6.21E-08	3.57E-08	2.85E-08	3.07E-08
**Ni**	3.70E-02	4.00E-02	2.00E-02	1.26E-07	1.36E-07	6.79E-08	1.79E-11	1.94E-11	9.70E-12
**Pb**	1.50E-01	1.30E-02	1.30E-01	2.91E-06	2.52E-07	2.52E-06	4.28E-10	3.71E-11	3.71E-10
**V**	5.00E-02	5.00E-02	3.00E-02	6.74E-07	6.74E-07	4.04E-07	7.13E-11	7.13E-11	4.28E-11
**Zn**	1.60E-01	1.90E-01	3.20E-01	3.62E-08	4.30E-08	7.24E-08	5.33E-12	6.32E-12	1.07E-11
HI	–	–	–	1.40E-05	1.23E-05	6.15E-06	8.61E-08	6.83E-08	5.64E-08

	**AD**_**inh**_			**AD**_**der**_			**AD**_**total**_		
	**urban**	**traffic**	**suburban**	**urban**	**traffic**	**suburban**	**urban**	**traffic**	**suburban**
**Al**	1.15E-11	1.31E-11	9.70E-12	3.33E-09	3.80E-09	2.81E-09	1.13E-07	1.29E-07	9.52E-08
**Fe**	6.78E-12	6.71E-12	4.57E-12	1.96E-09	1.94E-09	1.32E-09	6.65E-08	6.58E-08	4.48E-08
**As**	2.21E-13	2.57E-13	4.99E-14	6.41E-11	7.44E-11	1.45E-11	2.17E-09	2.52E-09	4.90E-10
**Cd**	1.50E-13	2.28E-13	1.43E-14	4.34E-11	6.62E-11	4.14E-12	1.47E-09	2.24E-09	1.40E-10
**Cr**	9.99E-13	7.85E-13	5.14E-13	2.89E-10	2.27E-10	1.49E-10	9.80E-09	7.70E-09	5.04E-09
**Cu**	1.28E-12	1.57E-12	2.64E-13	3.72E-10	4.55E-10	7.65E-11	1.26E-08	1.54E-08	2.59E-09
**Mn**	3.57E-13	2.85E-13	3.07E-13	1.03E-10	8.27E-11	8.89E-11	3.50E-09	2.80E-09	3.01E-09
**Ni**	2.64E-13	2.85E-13	1.43E-13	7.65E-11	8.27E-11	4.14E-11	2.59E-09	2.80E-09	1.40E-09
**Pb**	1.07E-12	9.27E-14	9.27E-13	3.10E-10	2.69E-11	2.69E-10	1.05E-08	9.10E-10	9.10E-09
**V**	3.57E-13	3.57E-13	2.14E-13	1.03E-10	1.03E-10	6.20E-11	3.50E-09	3.50E-09	2.10E-09
**Zn**	1.14E-12	1.36E-12	2.28E-12	3.31E-10	3.93E-10	6.62E-10	1.12E-08	1.33E-08	2.24E-08
	**HQ**_**der**_			**HQ**_**total**_			**RI**		
	**urban**	**traffic**	**suburban**	**urban**	**traffic**	**suburban**	**urban**	**traffic**	**suburban**
**Al**	–	–	–	–	–	–	–	–	–
**Fe**	–	–	–	–	–	–	–	–	–
**As**	1.80E-09	2.09E-09	4.06E-10	5.27E-06	6.11E-06	1.19E-06	2.32E-12	2.70E-12	5.25E-13
**Cd**	1.50E-08	2.28E-08	1.43E-09	1.44E-06	2.20E-06	1.37E-07	9.14E-13	1.39E-12	8.70E-14
**Cr**	1.66E-08	1.31E-08	8.56E-09	3.23E-06	2.54E-06	1.66E-06	4.09E-11	3.22E-11	2.11E-11
**Cu**	1.07E-10	1.31E-10	2.20E-11	3.06E-07	3.74E-07	6.28E-08	–	–	–
**Mn**	1.55E-10	1.24E-10	1.33E-10	1.08E-07	8.64E-08	9.29E-08	–	–	–
**Ni**	4.89E-11	5.28E-11	2.64E-11	1.26E-07	1.36E-07	6.79E-08	–	–	–
**Pb**	2.04E-09	1.77E-10	1.77E-09	2.91E-06	2.52E-07	2.52E-06	–	–	–
**V**	5.09E-09	5.09E-09	3.06E-09	6.79E-07	6.79E-07	4.07E-07	–	–	–
**Zn**	1.90E-11	2.26E-11	3.80E-11	3.62E-08	4.30E-08	7.25E-08	–	–	–
HI	4.09E-08	4.36E-08	1.54E-08	1.41E-05	1.24E-05	6.22E-06	–	–	–

**Table 5:** The adsorbed dose of exposure, hazard quotient, and Hazard Index of heavy metals via three main pathways in winter.

**Elements**	**Winter**			**AD**_**ing**_			**AD**_**inh**_		
	**urban**	**traffic**	**suburban**	**urban**	**traffic**	**suburban**	**urban**	**traffic**	**suburban**
**Al**	1.86E+00	1.29E+00	8.80E-01	1.26E-07	8.76E-08	5.98E-08	1.86E-11	1.29E-11	8.79E-12
**Fe**	1.31E+00	1.12E+00	7.10E-01	8.90E-08	7.61E-08	4.82E-08	1.31E-11	1.12E-11	7.09E-12
**As**	6.00E-02	3.00E-02	3.10E-02	4.07E-09	2.04E-09	2.11E-09	5.99E-13	3.00E-13	3.10E-13
**Cd**	3.40E-02	3.10E-02	2.00E-02	2.31E-09	2.11E-09	1.36E-09	3.40E-13	3.10E-13	2.00E-13
**Cr**	7.00E-02	7.00E-02	8.10E-02	4.75E-09	4.75E-09	5.50E-09	6.99E-13	6.99E-13	8.09E-13
**Cu**	3.40E-01	1.10E-01	4.00E-02	2.31E-08	7.47E-09	2.72E-09	3.40E-12	1.10E-12	3.99E-13
**Mn**	7.00E-02	3.00E-02	3.20E-02	4.75E-09	2.04E-09	2.17E-09	6.99E-13	3.00E-13	3.20E-13
**Ni**	3.20E-02	5.70E-02	2.80E-02	2.17E-09	3.87E-09	1.90E-09	3.20E-13	5.69E-13	2.80E-13
**Pb**	1.90E-01	2.00E-02	5.00E-02	1.29E-08	1.36E-09	3.40E-09	1.90E-12	2.00E-13	4.99E-13
**V**	4.00E-02	5.00E-02	2.00E-02	2.72E-09	3.40E-09	1.36E-09	3.99E-13	4.99E-13	2.00E-13
**Zn**	1.90E-01	1.20E-01	8.00E-02	1.29E-08	8.15E-09	5.43E-09	1.90E-12	1.20E-12	7.99E-13
**Elements**				**HQ**_**ing**_			**HQ**_**inh**_		
	**urban**	**traffic**	**suburban**	**urban**	**traffic**	**suburban**	**urban**	**traffic**	**suburban**
**Al**	1.86E+00	1.29E+00	8.80E-01	–	–	–	–	–	–
**Fe**	1.31E+00	1.12E+00	7.10E-01	–	–	–	–	–	–
**As**	6.00E-02	3.00E-02	3.10E-02	1.02E-05	5.09E-06	5.26E-06	1.50E-09	7.49E-10	7.74E-10
**Cd**	3.40E-02	3.10E-02	2.00E-02	2.31E-06	2.11E-06	1.36E-06	3.40E-10	3.10E-10	2.00E-10
**Cr**	7.00E-02	7.00E-02	8.10E-02	1.58E-06	1.58E-06	1.83E-06	2.44E-08	2.44E-08	2.83E-08
**Cu**	3.40E-01	1.10E-01	4.00E-02	5.77E-07	1.87E-07	6.79E-08	8.45E-11	2.73E-11	9.94E-12
**Mn**	7.00E-02	3.00E-02	3.20E-02	1.01E-07	4.33E-08	4.62E-08	4.99E-08	2.14E-08	2.28E-08
**Ni**	3.20E-02	5.70E-02	2.80E-02	1.09E-07	1.94E-07	9.51E-08	1.55E-11	2.76E-11	1.36E-11
**Pb**	1.90E-01	2.00E-02	5.00E-02	3.69E-06	3.88E-07	9.70E-07	5.42E-10	5.71E-11	1.43E-10
**V**	4.00E-02	5.00E-02	2.00E-02	5.39E-07	6.74E-07	2.69E-07	5.71E-11	7.13E-11	2.85E-11
**Zn**	1.90E-01	1.20E-01	8.00E-02	4.30E-08	2.72E-08	1.81E-08	6.32E-12	3.99E-12	2.66E-12
HI	–	–	–	1.91E-05	1.03E-05	9.92E-06	7.69E-08	4.71E-08	5.23E-08

**Elements**	**AD**_**inh**_			**AD**_**der**_			**AD**_**total**_		
	**urban**	**traffic**	**suburban**	**urban**	**traffic**	**suburban**	**urban**	**traffic**	**suburban**
**Al**	1.33E-11	9.20E-12	6.28E-12	3.85E-09	2.67E-09	1.82E-09	1.30E-07	9.03E-08	6.16E-08
**Fe**	9.34E-12	7.99E-12	5.06E-12	2.71E-09	2.32E-09	1.47E-09	9.17E-08	7.84E-08	4.97E-08
**As**	4.28E-13	2.14E-13	2.21E-13	1.24E-10	6.20E-11	6.41E-11	4.20E-09	2.10E-09	2.17E-09
**Cd**	2.43E-13	2.21E-13	1.43E-13	7.03E-11	6.41E-11	4.14E-11	2.38E-09	2.17E-09	1.40E-09
**Cr**	4.99E-13	4.99E-13	5.78E-13	1.45E-10	1.45E-10	1.67E-10	4.90E-09	4.90E-09	5.67E-09
**Cu**	2.43E-12	7.85E-13	2.85E-13	7.03E-10	2.27E-10	8.27E-11	2.38E-08	7.70E-09	2.80E-09
**Mn**	4.99E-13	2.14E-13	2.28E-13	1.45E-10	6.20E-11	6.62E-11	4.90E-09	2.10E-09	2.24E-09
**Ni**	2.28E-13	4.07E-13	2.00E-13	6.62E-11	1.18E-10	5.79E-11	2.24E-09	3.99E-09	1.96E-09
**Pb**	1.36E-12	1.43E-13	3.57E-13	3.93E-10	4.14E-11	1.03E-10	1.33E-08	1.40E-09	3.50E-09
**V**	2.85E-13	3.57E-13	1.43E-13	8.27E-11	1.03E-10	4.14E-11	2.80E-09	3.50E-09	1.40E-09
**Zn**	1.36E-12	8.56E-13	5.71E-13	3.93E-10	2.48E-10	1.65E-10	1.33E-08	8.40E-09	5.60E-09
**Elements**	**HQ**_**der**_			**HQ**_**total**_			**RI**		
	**urban**	**traffic**	**suburban**	**urban**	**traffic**	**suburban**	**urban**	**traffic**	**suburban**
**Al**	–	–	–	–	–	–	–	–	–
**Fe**	–	–	–	–	–	–	–	–	–
**As**	3.48E-09	1.74E-09	1.80E-09	1.02E-05	5.10E-06	5.27E-06	4.50E-12	2.25E-12	2.32E-12
**Cd**	2.43E-08	2.21E-08	1.43E-08	2.33E-06	2.13E-06	1.37E-06	1.48E-12	1.35E-12	8.70E-13
**Cr**	8.32E-09	8.32E-09	9.63E-09	1.62E-06	1.62E-06	1.87E-06	2.05E-11	2.05E-11	2.37E-11
**Cu**	2.02E-10	6.54E-11	2.38E-11	5.77E-07	1.87E-07	6.79E-08	–	–	–
**Mn**	2.17E-10	9.30E-11	9.92E-11	1.51E-07	6.48E-08	6.92E-08	–	–	–
**Ni**	4.23E-11	7.53E-11	3.70E-11	1.09E-07	1.94E-07	9.51E-08	–	–	–
**Pb**	2.58E-09	2.72E-10	6.79E-10	3.69E-06	3.88E-07	9.71E-07	–	–	–
**V**	4.08E-09	5.09E-09	2.04E-09	5.43E-07	6.79E-07	2.72E-07	–	–	–
**Zn**	2.26E-11	1.43E-11	9.51E-12	4.30E-08	2.72E-08	1.81E-08	–	–	–
HI	4.32E-08	3.78E-08	2.86E-08	1.93E-05	1.04E-05	1.00E-05	–	–	–
