# Supplementary material for: Health risk assessment of heavy metals on PM2.5 in Tehran air, Iran
Source: Data Brief. 2018 Jan 31;17:347–55. doi: 10.1016/j.dib.2018.01.018 (PMC5988319; doi:10.1016/j.dib.2018.01.018)
Supplement: Supplementary file 1 — Supplementary material. [file mmc1.docx]

Dear Professor,

Editor of **Data in Brief**

The authors of this article declare that they have no conflict of interests.

Anoushiravan MohseniBandpi^1^, Akbar Eslami^1^, Mansour Ghaderpoori^2, 3^, Abbas Shahsavani^1,4^, Ali Khani Jeihooni^5^, Afshin Ghaderpoury^6^, Abdolazim Alinejad^5*^.

1. Environmental and Occupational Hazards Control Research Center, Shahid Beheshti University of Medical Sciences, Tehran, Iran
2. Nutritional Health Research Center, Lorestan University of Medical Sciences, Khorramabad, Iran
3. Department of environmental health engineering, School of Health and Nutrition, Lorestan University of medical sciences, Khorramabad, Iran ( [mghaderpoori@gmail.com](mailto:mghaderpoori@gmail.com))
4. Department of Environmental Health Engineering, School of Public Health, Shahid Beheshti University of Medical Sciences, Tehran, Iran
5. Department of Public Health, Fasa University of Medical Sciences, Fasa, Iran
6. Students Research Committee, Shahid Beheshti University of Medical Sciences, Tehran, Iran ([ghaderpoury_a@yahoo.com](mailto:ghaderpoury_a@yahoo.com))

- **Corresponding author Contact email**: Abdolazim Alinejad, Mansour Ghaderpoori ([azimalinejad@gmail.com](mailto:azimalinejad@gmail.com), mghaderpoori@gmail.com)
